# The role of the somatosensory system in the feeling of emotions: a neurostimulation study

**DOI:** 10.1093/scan/nsae062

**Published:** 2024-09-13

**Authors:** Michelle Giraud, Amir-Homayoun Javadi, Carmen Lenatti, John Allen, Luigi Tamè, Elena Nava

**Affiliations:** Department of Psychology, University of Milano-Bicocca, Milano 20126, Italy; School of Psychology, University of Kent, Canterbury CT2 7NZ, United Kingdom; Psychology Department and NeuroMi, Milan Centre of Neuroscience, University of Milano-Bicocca, Milan 20126, Italy; School of Psychology, University of Kent, Canterbury CT2 7NZ, United Kingdom; School of Rehabilitation, Tehran University of Medical Sciences, Tehran 1416634793, Iran; School of Psychology, University of Kent, Canterbury CT2 7NZ, United Kingdom; School of Psychology, University of Kent, Canterbury CT2 7NZ, United Kingdom; School of Psychology, University of Kent, Canterbury CT2 7NZ, United Kingdom; Department of Psychology, University of Milano-Bicocca, Milano 20126, Italy; Psychology Department and NeuroMi, Milan Centre of Neuroscience, University of Milano-Bicocca, Milan 20126, Italy

**Keywords:** transcranial alternating current stimulation (tACS), emotion perception, emotion generation, embodied emotion, somatosensory primary system

## Abstract

Emotional experiences deeply impact our bodily states, such as when we feel ‘anger’, our fists close and our face burns. Recent studies have shown that emotions can be mapped onto specific body areas, suggesting a possible role of the primary somatosensory system (S1) in emotion processing. To date, however, the causal role of S1 in emotion generation remains unclear. To address this question, we applied transcranial alternating current stimulation (tACS) on the S1 at different frequencies (beta, theta, and sham) while participants saw emotional stimuli with different degrees of pleasantness and levels of arousal. Results showed that modulation of S1 influenced subjective emotional ratings as a function of the frequency applied. While theta and beta-tACS made participants rate the emotional images as more pleasant (higher valence), only theta-tACS lowered the subjective arousal ratings (more calming). Skin conductance responses recorded throughout the experiment confirmed a different arousal for pleasant versus unpleasant stimuli. Our study revealed that S1 has a causal role in the feeling of emotions, adding new insight into the embodied nature of emotions. Importantly, we provided causal evidence that beta and theta frequencies contribute differently to the modulation of two dimensions of emotions—arousal and valence—corroborating the view of a dissociation between these two dimensions of emotions.

## Introduction

The role of the human body in generating and perceiving emotional subjective experiences has been discussed over a century, with views placing the body either as a direct trigger of any emotional reaction (James–Lange theory) or as a coactive part during any emotional experience (Cannon–Bard theory) (James 1884, Cannon 1927, Darwin 1965).

New lifeblood of this debate has come from the idea of placing emotions within an embodied cognition perspective, highlighting the involvement of sensory, perceptual, somatosensory, motor, and linguistic representations not only when we experience emotions but also when we think about them (Niedenthal et al. 2005, Niedenthal 2007, Niedenthal and Maringer 2009, Winkielman et al. 2015, Carr et al. 2018). Specifically, in this view, the subjective perception of bodily internal states, external stimulus, and conceptual knowledge about the world underlie any information processing that contributes to forming different mental states. Among those elements, somatosensory representations specifically have played a crucial role in emotion processing. Indeed, an embodied perspective considers any cognitive process as deeply rooted in the body’s interactions with the world (Wilson 2002, Foglia and Wilson 2013).

Evidence for this perspective comes from studies that have demonstrated, e.g. that perceiving and discriminating emotional expressions, as opposed to neutral ones, triggers embodied resonance in sensorimotor regions, which implies re-enacting the visceral, somatic, proprioceptive, and motor patterns associated with the observed expressions (Niedenthal 2007, Keysers and Gazzola 2009, Rudrauf et al. 2009, Keysers et al. 2010, Gallese and Sinigaglia 2018).

The tight relationship between emotions and body is further evidenced by studies in which an artificial manipulation of bodily feedback or facial expression and body postures can alter emotional attribution (Price and Harmon‐Jones 2015). It has been observed that perceived emotional intensity/salience of neutral faces increases when accompanied by false feedback of increased heart rate (Gray et al. 2007), and the more accurately participants can track heart rate, the stronger is the observed link between heart rate changes and subjective ratings of arousal (but not valence) of emotional images (Dunn et al. 2010). For example, the processing of brief fear stimuli is selectively gated by their timing in relation to individual heartbeats: fearful faces are detected more easily and rated as more intense at systole than at diastole (i.e. when the heart muscles contract) (Garfinkel et al. 2014). Even some artificial manipulation of organ activity can induce emotions; for instance, intravenous administration of cholecystokinin can provoke panic attacks (Harro and Vasar 1991, Rehfeld 2021) and interfere with unpleasant tastes and smells, eliciting adverse somatovisceral responses (Wicker et al. 2003, Jabbi et al. 2007).

The specific involvement of the somatosensory cortices in perceiving and discriminating emotion is also supported by functional magnetic resonance imaging (fMRI) studies (Adolphs et al. 2000, Adolphs 2002, Nummenmaa et al. 2008, Straube and Miltner 2011, Terasawa et al. 2013, Giraud et al. 2024), as well as other neuroimaging techniques (Damasio et al. 2000, Rudrauf et al. 2009, Sel et al. 2014, 2020). For example, fMRI studies have shown that perceiving vocal and facial expressions of emotion yields haemodynamic activity in the right somatosensory cortex that discriminates among emotion categories (Kragel and LaBar 2016). In a positron emission tomography study, Damasio and colleagues (Damasio et al. 2000) showed that the process of feeling emotions involves the engagement of brain regions required in the homeostasis of bodily states, such as the somatosensory areas and upper brainstem nuclei. Moreover, other regions such as the amygdala, the anterior insula, the anterior cingulate cortex, the primary somatosensory cortex, and the medial prefrontal cortex (PFC) have shown stronger activation for threatening images compared to neutral ones (Straube and Miltner 2011).

Further research using transcranial magnetic stimulation (TMS) (Pourtois et al. 2004, Pitcher et al. 2008) and lesion methods (Adolphs et al. 2000, Atkinson and Adolphs 2011) has also demonstrated the contribution of nonvisual cortical areas, e.g. right somatosensory cortices, during the early stage of facial emotion expression recognition. These findings align with embodied cognition theories, claiming that recognizing facial expressions also requires the internal simulation of the somatovisceral responses associated with the perceived facial expression. Even electroencephalography (EEG) studies have revealed that somatosensory areas are involved in facial emotion recognition and understanding others’ emotional states (Sel et al. 2014, 2020). An EEG study by Sel et al. (2014) showed that the somatosensory cortex plays an important role in face emotion processing (i.e. happiness and fear) over and above any visual carryover activity. Indeed, emotional face processing influenced somatosensory responses to both face and finger tactile stimulation, implying a wider process that includes nonfacial cortical representations, too, and providing neural evidence for emotional expression embodiment beyond visual analysis of emotions.

Thus, the literature mentioned so far suggests a tight relationship between emotions, the body, and the brain, particularly implying the involvement of the somatosensory cortices in processing emotional experiences. However, whether there is a causal role of the somatosensory cortices in emotion perception and generation remains to be deepened. Such knowledge is pivotal to better understanding the physiological theories of emotions, as well as aligning with embodied cognition theories.

Here, we investigated the causal link between generated emotions and the somatosensory system by temporarily altering brain oscillations around the primary somatosensory cortices (left and right S1) using transcranial alternating current stimulation (tACS). tACS is a relatively new electrical brain stimulation technique that delivers alternating electric currents directly to the scalp, frequency-dependent manner and modulating the activity of a specific targeted area of the brain (Paulus 2011, Antal and Paulus 2013). tACS is considered to entrain endogenous brain rhythm by modifying underlying membrane potential (Chan and Nicholson 1986) and has been demonstrated to influence both frequency (Helfrich et al. 2014) and synchronization (Polanía et al. 2012), even though the underlying neural mechanism and the on-set/off-set effects are not completely understood. Contrary to other neuroimaging techniques (e.g. fMRI), which mostly can provide correlational accounts between brain activity and behaviour or cognition, tACS can be used to assess a direct causal relationship between a specific portion of the brain and a certain cognitive process, processing emotions in our case. For instance, it has been found that the TMS-induced phosphene threshold is reduced by 20 Hz tACS stimulation during 5–8 min per session (i.e. increased visual cortex excitability) (Kanai et al. 2010). This suggests that tACS can be used to establish a causal relationship between rhythmic cortical processes and their functions.

To date, tACS has been mainly used in research settings to modulate primary motor cortex (Feurra et al. 2011, Pollok et al. 2015, Sugata et al. 2018) or higher-order functions, such as memory and executive processing (Sela et al. 2012a, Pahor and Jaušovec 2014, Jaušovec and Jaušovec 2014a). There is still little research that has investigated the possible modulatory effects of tACS stimulation on the primary somatosensory cortex by demonstrating statistically robust efficacy, and, to the best of our knowledge, most studies have hypothesized a cortical entrainment due to neurostimulation based primarily on the observation of changes in behavioural measures (Gundlach et al. 2016a, Wittenberg et al. 2019, Manzo et al. 2020a). For example, tACS applied over S1 has proved to induce tactile sensations in a frequency-dependent manner (Feurra et al. 2011a) and is able to modulate early somatosensory information processing at the S1 level (Fabbrini et al. 2022). This suggests that this technique can be effective at directly interacting with the cortical activity and can successfully modulate the somatosensory cortex (Tamè and Holmes 2023).

In the present study, the targeted area (i.e. somatosensory primary cortex) and frequencies of tACS were chosen based on a careful inspection of the previous literature on the role of neural oscillation in somatosensation and emotion processes. Thus, we decided to specifically target two frequencies: beta frequency (12.5–30 Hz) for somatosensation and theta frequency (4–8 Hz) for emotion processes. In particular, beta frequency has proved to be a sort of ‘natural frequency’ reflecting sensorimotor activity. Previous electro- or magnetoencephalography studies using a correlative approach have shown that beta neural oscillations emerging in the sensorimotor area can influence the regulation of motor response vigour (e.g. slowness of movements), and they are involved in the motor control of repetitive finger movements (Guerra et al. 2018, Uehara et al. 2023). tACS applied at 20 Hz can slow down voluntary movements (Gilbertson et al. 2005, Pogosyan et al. 2009, Rosanova et al. 2009, Joundi et al. 2012, Wach et al. 2013) by disrupting sensorimotor integration. tACS over the somatosensory cortex has shown encouraging effects on inducing tactile sensation and increased tactile discrimination with the involvement of alpha, high gamma, and beta frequencies (Feurra et al. 2011a, Gundlach et al. 2017, Saito et al. 2021). Despite this, however, it is important to note that results are still contradictory regarding the robustness of the effects of tACS on S1 (Manzo et al. 2020a). Regarding the emotion domain, beta activity has been poorly explored in response to emotional stimuli, and the results are still contradictory (Balconi and Pozzoli 2007, Güntekin and Basar 2007, Knyazev et al. 2008, Okazaki et al. 2008). In broad terms, beta activity appears to be linked to changes in sensory processing and oscillatory events within the sensorimotor cortex of humans. This observation suggests that beta activity may concurrently impact cortical sensory processing, motor output, and sensory–motor interactions (Lalo et al. 2007, Haegens et al. 2011, Baumgarten et al. 2015).

On the contrary, theta rhythm does not seem to have a major role in somatosensory cortical processing (Cheyne 2013); in turn, it seems to be involved in attentive, cognitive, and emotional processes (Balconi and Pozzoli 2009, Ertl et al. 2013). Indeed, different studies suggest the importance of low-frequency oscillations in delta, theta, and alpha bands in the context of emotion processes, e.g. emotion regulation (ER) and negative stimuli processing. Theta oscillations were typically related to emotional regulation and involved in both dimensions of emotions, i.e. ‘affective valence’ and ‘valence’. For example, a study by Aftanas et al. (2001) revealed a different modulation of the theta band caused by the ‘affective valence’ of the emotional picture presented and an increase in theta activity in the amygdala caused by the ‘arousal’ of the emotional stimuli experienced (e.g. fear; Pare 2003, Lesting et al. 2011, Ertl et al. 2013). Moreover, recent research showed that event-related theta band (3–7 Hz) responds to prolonged visual emotional stimulation (e.g. theta responds to the emotional significance of the face in processing facial expression; Sollfrank et al. 2021) and responds to negative stimuli within the right side of the scalp (Krause et al. 2000, Güntekin and Basar 2007, Knyazev 2007, Balconi and Lucchiari 2008, Balconi and Pozzoli 2009).

In sum, beta and theta frequencies were chosen to possibly differentiate the contribution of somatosensory and emotional processes in the somatosensory cortices when exposed to emotional scenes [e.g. stimuli from International Affective Pictures System (IAPS)] that activate feelings of emotions. Based on the hypothesis that cortical regions that commonly respond to tactile and more generally bodily sensations (e.g. somatosensory primary cortices) could contribute significantly to subjective emotion experiences, we asked participants to rate emotional scenes while stimulated with tACS, assuming that if the S1 is involved in emotional processing, participants would rate the valence (i.e. unpleasantness/pleasantness) and/or arousal (i.e. exciting/calming) of those scenes differently before stimulation compared to during stimulation.

Moreover, skin conductance responses (SCRs) were recorded throughout the experiment to obtain a measure of the general physiological arousal of the participants and to control that emotional stimuli were really felt as more arousing than neutral ones (i.e. significant skin conductance response for pleasant and unpleant stimuli than for neutral stimuli). We did not expect tACS to modulate autonomic activity, as previous studies did not find a statistically robust effect on SCRs (Jensen et al. 2014, May et al. 2021). On the contrary, and in line with an embodied view, we expected to find changes in the processing of emotions when S1 was stimulated bilaterally. Specifically, we expected to find a modulatory effect of beta and theta stimulations on participants’ ratings compared to when a sham stimulation is applied. Selective influence of either beta or theta frequency (i.e. more influence on participants’ rating of one of the two frequencies) would provide evidence of the weight of either the somatosensory or emotional component in the processing of emotions.

## Materials and methods

### Participants

Sixty participants of both genders (*N* = 40 females, age range: 20–35 years; mean ± SD: 24 ± 4) participated in the study. The sample size was chosen following *a priori* sample size calculation for a repeated measures analysis of variance (rmANOVA, 0.25 effect size, *α* err. prob. 0.05, and power 0.95, *N* = 36) (Faul et al. 2007, 2009). Participants were recruited among the student population of the University of Kent. All participants gave informed consent prior to testing and were informed about the experimental procedure. The study was conducted in accordance with the Declaration of Helsinki (Anon 2013) and approved by the local ethical committee of the School of Psychology at the University of Kent (Protocol number: 7661).

Participants were pseudo-randomly assigned to three different stimulation conditions associated with three experimental groups of 20 participants each, attempting to balance participants for age and gender.

### Stimuli and procedure

This experiment was designed as a double-blind, between-subjects, sham-controlled trial study to avoid awareness of condition assignments and learning effects. One researcher performed the study (G.M.), whereas a second researcher set the tACS parameters and assigned participants randomly to one of the three stimulation frequencies (i.e. beta, theta, or sham) (L.C.). Participants were pseudo-randomly assigned to three different stimulation conditions:

- Beta-tACS group received active neurostimulation applied in the beta frequency range (20.11 Hz, cycle 18 000, fade in 200 cycles).
- Theta-tACS group received active neurostimulation applied in the theta frequency range (6.00 Hz, cycle 5400, fade in 60 cycles).
- Sham-tACS group, or control group, in which participants received only a few seconds of neurostimulation, so the procedure mimicked the characteristics of active stimulation to achieve blinding integrity (i.e. maximum 6–8 s of active stimulation; afterwards, the device was turned off).

Participants had no history of neurological or psychiatric disorders and normal or corrected-to-normal vision. Before testing, all participants were assessed on the Edinburgh Handedness Inventory (Oldfield 1971); most of them were right-handed, except five who were left-handed.

They also answered the Multidimensional Assessment of Interoceptive Awareness scale, Version 2 (MAIA-2, Mehling et al. 2018), to examine whether participants might differ in their interoceptive awareness, i.e. higher scores indicate beneficial self-reported interoception. MAIA-2 is an 8-subscale state-trait self-report questionnaire used to measure multiple dimensions of interoception, conceived as the nervous system’s process of perceiving, interpreting, and integrating signals from within the body (Eggart et al. 2021). Low interoceptive awareness has been linked to issues with emotion awareness and modulation (Price and Hooven 2018). The results from the MAIA-2 focus on the individual scale scores as a total score is not meaningful (Mehling et al. 2018). Participants’ scores and data analysis can be found in the Supplementary materials (see Supplemental Materials, https://osf.io/a5c93/).

**Figure 1.**
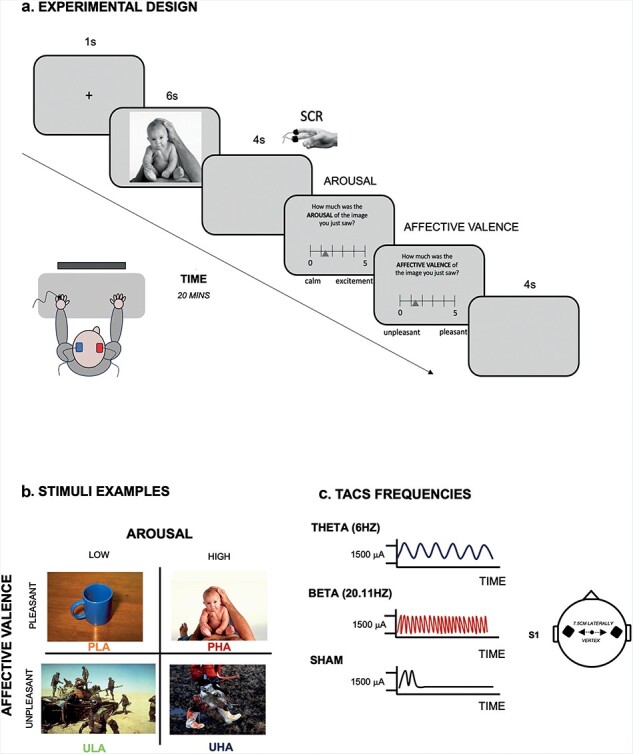
The figure display the experimental design (panel A), the stimuli and conditions (panel B), and the frequencies used during tACS stimulatuon (panel C).

Stimuli were selected from the IAPS (Lang et al. 1999), according to the arousal norm rating (i.e. we selected the most arousing images) as a function of gender. In other words, we created two subsets of stimuli fitting best to male and female participants. Then, images were divided into 4 categories, 15 per category: pleasant high arousal (PHA), pleasant low arousal (PLA), unpleasant high arousal (UHA), and unpleasant low arousal (ULA) (see Table 1 in the Supplementary Material for the list of images used, Fig. 1b for stimuli example). SCR was recorded continuously during the Intensity Rating task. The participants’ electrodermal activity was recorded using a biological signal amplifier (BIOPAC system MP35), and an optical connection was used to connect the amplifier to the computer. The signal was sampled at 1000 Hz (signal parameter set at 5 mho/V). The reference electrode was placed on the left hand’s ring finger, and a trigger was assigned to each category of images (i.e. four triggers in total). Data were analysed using MATLAB 2022b (Mathworks, USA). The data were split over different stimulus and stimulation conditions. The data of interest were extracted between −1 s and +20 s from the onset of the stimulus presentation. The data were baseline-corrected using the average of the activity between −1.5 s and −1.0 s of the onset of the stimulus presentation. Subsequently, the average of this activity was used for data analysis. Three participants were excluded from the analysis due to the poor quality of the signals recorded (final sample *N* = 57). Raw data and MATLAB script can be found at the following OSF link: https://osf.io/a5c93/.

**Table 1.** Three experimental groups.

Group	Total	Male	Female
Beta-tACS	20	7	13
Theta-tACS	20	7	13
Sham-tACS	20	6	14
Mean age	24.10	28.15	22.08
s.d. age	4.42	3.94	3.06

#### Intensity rating task

The intensity rating task was divided into two blocks, with at least 3 min breaks between those, and participants viewed a total of 120 emotional images (60 per block):

- First block, labelled as ‘before-tACS’, was carried out without using neurostimulation (i.e. tACS).
- Second block, labelled as ‘during-tACS’, was carried out using neurostimulation (i.e. tACS) under three different stimulation conditions (i.e. beta frequency, theta frequency, or sham mode; depending on the participant’s allocated group).

Each IAPS image was displayed on the screen for 6 s; afterwards, an interstimulus interval (ISI) of 4 s was delivered to allow the SCR from the previous trial to return to baseline (Sjouwerman and Lonsdorf 2019). A grey screen was displayed during the ISI. Afterwards, participants saw two intensity rating questions on the screen: ‘How much was the Arousal/Affective Valence of the image you just saw? Use the mouse to move along the continuous scale in the middle of the screen and rate it. A red triangle will appear to indicate your choice.’ They were instructed to rate, as fast as possible, the Affective Valence and Arousal within a continuous scale of 0–5 presented on the screen, e.g. from ‘very unpleasant/very calming’ ‘0’, to ‘very pleasant/very exciting’ ‘5’ (Fig. 1a shows an example of a typical trial). Before starting the experiment, all participants were explained the meaning of Affective Valence and Arousal to make sure they understood the assignment correctly. This is the text included in the information sheet: the Affective Valence scale covers the direction of the feeling or emotion. This ascertains if the feeling evoked by the image is positive or negative without mentioning how evocative it is. Instead, the Arousal scale refers to the intensity of the emotion experienced in response to the image. It captures information about whether the material is calming or exciting without referencing the image’s positive or negative nature.

### Transcranial alternating current stimulation

tACS was applied through a pair of saline-soaked surface sponge electrodes connected to a battery-driven alternated current stimulator (DC-Stimulator PLUS, NeuroConn GmbH, Ilmenau, Germany). We used 3 × 4 cm electrodes applied bilaterally to the primary somatosensory cortices (left and right S1), which were 7.5 cm laterally from the vertex (Holmes and Tamè 2019, Holmes et al. 2019). A tACS fixed intensity of 1500 A was applied during active stimulation for 20 min, and the impedance between the electrodes was kept <8 kΩ to avoid any painful sensations.

At the end of the tACS stimulation, participants were asked to describe their subjective experience, indicating any physical sensation experienced. All responses generally pointed to the same physical sensations: an initial itching sensation on the skull where the patches were applied or no physical sensation at all. No side effects (e.g. visual flickering or painful sensations) were observed across participants. This survey was a modified version of the questionnaire developed by Fertonani et al. (2015) following their items: at the end of tACS stimulation, participants were asked whether they experienced any physical sensations (e.g. itches, pain, burning, and heat) and for how long, and whether they believe to have received a real or placebo stimulation. Yet, the participants’ responses indicated that the physical feedback they experienced was never described as painful or annoying to the point of discontinuing the experiment, and they were unable to recognize which of the three experimental groups they belonged to.

### Statistical analysis

#### Behavioural analysis of subjective ratings

Subjective affective valence and arousal rating variables were analysed by rmANOVA using Jamovi software v2.3.28 (2023, https://www.jamovi.org). We performed two rmANOVAs, separately for Affective Valence and Arousal dimensions, with Pleasantness (pleasant versus unpleasant), Intensity (high versus low), and Time (before versus during stimulation) as within-subject factors, and Stimulation (beta, theta, and sham) as a between-subject factor. Significant interactions were further explored using Tukey’s post-hoc tests. The two separate ANOVAs were motivated by our strong predictions about the different effects of tACS depending on stimuli emotion dimensions based on robust and consistent literature demonstrating that Affective Valence and Arousal should not only be considered as separable dimensions in the study of emotions but also appear to be related to different brain circuits (Anderson et al. 2003, Small et al. 2003, Anders et al. 2004, Dolcos et al. 2004, Lewis et al. 2007, Nielen et al. 2009, Colibazzi et al. 2010, Kron et al. 2015).

#### SCR analysis

Similarly, SCR data were analysed by rmANOVA using Jamovi software, with Pleasantness, Intensity, and Time as within-subject factors and Stimulation as between-subject factor.

## Results

### Effects of tACS on behavioural subjective ratings

The ANOVA performed on Affective Valence revealed a series of main effects and interactions. The main effects included: Pleasantness, *F*(1,57) = 648, *P *<.001, *η*^2^ = 0.81; Intensity, *F*(1,57) = 42.3, *P *<.001, *η*^2^ = 0.011; and Time, *F*(1,57), *P *<.001, *η*^2^ = 0.002. There was no main effect of Stimulation, *F*(2,57) = 0.85, *P *= .433, *η*^2^ = .001.

The analysis also revealed a series of interactions, such as Pleasantness and Intensity, *F*(2,57) = 237, *P *<.001, *η*^2^ = 0.05. As expected, this was caused by higher subjective ratings for pleasant high images (i.e. PHA) compared to unpleasant high images (i.e. UHA) [PHA: *M* = 3.72, SE = 0.08; UHA: *M* = 0.66, SE = 0.06, *t*(57) = 26.65, *P *<.001, *d_z_ *= 6.03] and higher subjective rating for pleasant low images (i.e. PLA) compared to unpleasant low images (i.e. ULA) [PLA: *M* = 3.42, SE = 0.07; ULA: *M* = 1.42, SE = 0.11, *t*(57) = 10.26, *P *<.001, *d_z_ *= 4].

There was a significant interaction between Intensity and Time, *F*(2,57) = 8.1, *P *= .006, *η*^2^ = 0, caused by higher subjective ratings for PHA and UHA images within ‘During’ compared to ‘Before’ neurostimulation [High-Before: *M* = 2.15, SE = 0.03; High-During: *M* = 2.23, SE = 0.03, *t*(57) = −3.65, *P *= .003, *d_z_* = 0.1], and higher subjective rating for PLA and ULA images within ‘During’ compared to ‘Before’ neurostimulation [Low-Before: *M* = 2.38, SE = 0.04; Low-During: *M* = 2.57, SE = 0.04, *t*(57) = −6.58, *P *<.001, *d_z_ *= 0.2].

Finally, there was an interaction between Time and Stimulation, *F*(2,57) = 12.53, *P *<.001, *η*^2^ = 0.001. Post-hoc analysis revealed that participants showed higher ratings for Affective Valence across all image categories following beta stimulation [Before-Beta: *M* = 2.25, SE = 0.05; During-Beta: *M* = 2.48, SE = 0.05, *t*(57)= −8.3, *P *<.001, *d_z_ *= 0.2] and theta stimulation [Before-Theta: *M* = 2.28, SE = 0.05; During-Theta: *M* = 2.42, SE = 0.05, *t*(57)= −5, *P *<.001, *d_z_ *= 0.1] compared to sham mode [Before-Sham: *M* = 2.27, SE = 0.05; During-Sham: *M* = 2.30, SE = 0.05, *t*(57)= −1, *P *= .91].

The ANOVA performed on the Arousal dimension revealed a series of main effects and interactions. The main effects included: Pleasantness, *F*(1,57) = 90, *P *<.001, *η*^2^ = 0.2; Intensity, *F*(1,57) = 300.23, *P *<.001, *η*^2^ = 0.33. There was no main effect of Stimulation, *F*(2,57) = 0.1, *P *= 1, *η*^2^ = 0.001.

The analysis also revealed a series of interactions, such as Pleasantness and Intensity, *F*(2,57) = 8.75, *P *= .004, *η*^2^ = 0.005. As expected, this was caused by higher subjective ratings for unpleasant high images (i.e. UHA) compared to pleasant high images (i.e. PHA) [UHA: *M* = 3.64, SE = 0.11; PHA: *M* = 2.79, SE = 0.11, *t*(57)= −6.20, *P *<.001, *d_z_ *= 1] and higher subjective rating for unpleasant low images (i.e. ULA) compared to pleasant low images (i.e. PLA) [ULA: *M* = 2.41, SE = 0.10; PLA: *M* = 1.22, SE = 0.10, *t*(57) = 11.20, *P *<.001, *d_z_ = *1.42].

There was a significant interaction between Intensity and Stimulation, *F*(2,57) = 3.74, *P *= .03, *η*^2^ = 0.008, caused by higher subjective ratings in Arousal dimension for high-image categories (i.e. PHA and UHA) compared to low-image categories (i.e. PLA and ULA) for all the three stimulation groups [Sham-High-image: *M* = 3.18, SE = 0.16; Sham-Low-image: *M* = 1.92, SE = 0.15, *t*(57) = 9.02, *P *<.001, *d_z_ *= 1.23. Theta-High-image: *M* = 3.33, SE = 0.16; Theta-Low-image: *M* = 1.62, SE = 0.15, *t*(57) = 12.23, *P *<.001*, d_z_ *= 2. Beta-High-Image: *M* = 3.15, SE = 0.16; Beta-Low-Image: *M* = 1.92, SE = 0.15, *t*(57) = 8.57, *P *<.001, *d_z_ *= 1.24].

Finally, there was an interaction between Time and Stimulation, *F*(2,57) = 6.9, *P *<.002, *η*^2^ = 0.003. Post-hoc analysis revealed that participants showed higher ratings for Arousal across all image categories following theta stimulation [Before-Theta: *M* = 2.56, SE = 0.14; During-Theta: *M* = 2.38, SE = 0.14, *t*(57) = 3.3, *P *= .022, *d_z_ *= 0.14] compared to beta stimulation [Before-Beta: *M* = 2.51, SE = 0.14; During-Beta: *M* = 2.56, SE = 0.14, *t*(57) = −0.84, *P *= .96] and sham mode [Before-Sham: *M* = 2.50, SE = 0.14; During-Sham: *M* = 2.60, SE = 0.14, *t*(57) = −1.62, *P *= .6].

**Figure 2.**
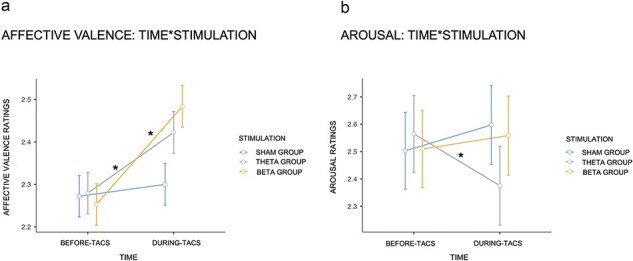
Interaction effects. (a) For all categories of stimuli, participants rated them as more positive (i.e. regarding the Affective Valence dimension) following beta and theta stimulations, showing an enhancement in subjective ratings following those neurostimulations, but not the sham mode. (b) For all categories of stimuli, participants rated them as more calming (i.e. regarding the Arousal dimension) following theta stimulation, showing a decrease in how participants rated the arousing of images Error bars represent the standard error of the mean (±SEM).

Overall, we can observe effects on behaviour (i.e. subjective evaluations) for neurostimulation but not for the sham mode, in which beta and theta frequency seem to influence the Affective Valence dimension of images, making all four categories of stimuli perceived and subsequently rated as more pleasant (i.e. PHA, PLA, UHA, and ULA). Moreover, theta frequency seems to influence the perceptual arousal of images, showing a calming effect on all four categories of stimuli (i.e. only during theta-tACS stimulation participants rated emotional stimuli as less arousing than they did before neurostimulation) (see Fig. 2a and b).

### Effects of tACS on SCR

**Figure 3.**
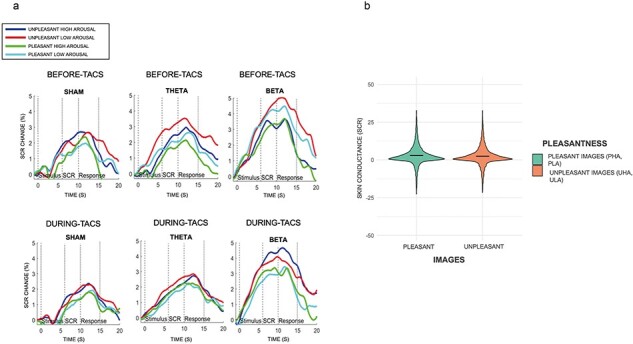
(a) Visual graphs of SCR data processing. Graphs visually show any possible changes in participants’ SCRs in the two experimental conditions, before and during tACS stimulations, for all image categories (UHA, ULA, PHA, and PLA). (b) The main effect of pleasantness in physiological data (i.e. SCR): we can only observe a difference in participants’ physiological responses between pleasant images (PHA and PLA) compared to unpleasant images (UHA and ULA).

The analyses revealed a main effect of image Pleasantness, i.e. unpleasant images (*M* = 2.88, SE = 0.4, *P *= .001) showed higher SCR responses than pleasant ones [*M* = 2.25, SE = 0.32; *F*(1,54) = 11.67, *P *= .001, *η*^2^ = 0.009; see Fig. 3a and b]. No other main effects on the SCR data were found for Time [e.g. before versus during stimulation; *F*(1,54) = 0.91, *P *= .344, *η*^2^ = 0.001] or Intensity [e.g. high versus low; *F*(1,54) = 1.20, *P *= .277, *η*^2^ = 0.001]. No significant differences between Stimulation (e.g. sham, theta and beta) were found [*F*(2,54) = 2.44, *P *= .1, *η*^2^ = 0.055].

## Discussion

This study investigated the causal role of the somatosensory system—with a focus on the primary somatosensory cortex—in the perception and generation of emotions using a neurostimulation approach. By altering the activity of the somatosensory cortex, we aimed to document to what extent S1 might be implicated in emotional processing.

The most important finding is that temporarily altering the brain oscillations in the somatosensory cortices altered the subjective perception of emotional images and the subjective feeling of emotions associated with them (i.e. behavioural responses to emotional Affective Valence and Arousal dimensions). Indeed, both beta and theta frequencies modulated participants’ ratings but in a slightly different fashion. In other words, both beta and theta were found to be involved in the Affective Valence of emotion and to modulate the perception of pleasant (i.e. PHA and PLA) and unpleasant (i.e. UHA and ULA) images, particularly increasing the positivity associated with them. On the other hand, theta modulated the aspects related to the Arousal of emotions, lowering the subjective arousal associated with all image categories. Thus, it appears that, depending on the frequency, the somatosensory system contributes to the perceptual judgement of emotional Affective Valence and Arousal.

Affective Valence and Arousal are considered the two primary dimensions describing the affective experience (Russell 1980), and although it is intrinsically difficult to dissociate the neural coding of these affective dimensions in the human brain, recent studies have shown that the affective representations of Arousal and Valence may draw upon dissociable neural substrates (Anderson et al. 2003, Small et al. 2003, Lewis et al. 2007, Kron et al. 2015, Gerdes et al. 2010). It has been proposed that neural circuits mediate various facets of emotional responses. For instance, behavioural reactions associated with Affective Valence exhibit a positive correlation with the amygdala and insular cortex activity, whereas Arousal responses display a correlation with thalamic and frontomedial activity (Anders et al. 2004); furthermore, peripheral physiological responses (e.g. SCR and startle reflex) also appear to contribute to the functional specialization of brain structures (Mahon and Cantlon 2011, Critchley and Harrison 2013). In particular, it has been shown that an increase in the startle reflex aligns with amygdala activity, whereas SCRs correspond to frontomedial activity; in contrast, Affective Valence and Arousal responses are associated with insular and thalamic activity, respectively (Anders et al. 2004). This divergence can also be observed in patients with brain-focal lesions in different brain areas, where dissociations between peripheral physiological and verbal responses emerged (Bechara et al. 1995, Peper and Irle 1997, Williams et al. 2001, Sánchez-Navarro et al. 2005, Soussignan et al. 2005). Furthermore, in the chemosensory domain, a dissociation between these two emotion dimensions can be observed, in which the cerebellum, pons, middle insula, and amygdala process arousal irrespective of valence, whereas the anterior insula-operculum and orbitofrontal cortex process valence-specific responses (Anderson et al. 2003, Small et al. 2003, Lewis et al. 2007).

A dissociable effect of Arousal and Affective Valence can be observed in the PFC activity indexing emotional evaluation and subsequent memory, too, in which dorsomedial PFC activity is sensitive to arousal dimension, while ventromedial PFC activity is sensitive to positive valence (Dolcos et al. 2004). This gives rise to the hypothesis that diverse facets of emotional responses are governed by distinct neural circuits. Such a proposition aligns with psychological theories positing the foundational structure of emotion, contending that emotional experience arises from the activation of distinct dimensions representing emotional arousal and valence (Russell 1980, Lang et al. 1993, Barrett and Russell 1999, Britton et al. 2006). In accordance with previous research, our results show a similar dissociation between valence and arousal dimensions, suggesting that neurostimulation (i.e. tACS) of the primary somatosensory cortices may affect differential aspects of emotional processes, e.g. the participants’ verbal responses, following the use of distinct frequencies (i.e. beta and theta frequencies).

All frequency bands of human cortical activity may have some functional significance, and each frequency band could be linked with specific processes (Abhang et al. 2016, Klimesch 2018). Indeed, beta and theta frequencies can be related to differential brain functions and outcome behavioural responses. Theta is associated with several brain functions, ranging from emotion-related behaviour and exploratory locomotion to high cognitive processes (e.g. working memory or executive processing; Sela et al. 2012a, Jaušovec and Jaušovec 2014a) (Korotkova et al. 2018). In the domain of emotion processes, theta has been linked to the perception and evaluation of arousing and salience emotional cues (Aftanas et al. 2001a, Balconi and Pozzoli 2009, Knyazev et al. 2009), in which, e.g. affective valence discrimination is associated with the early time-locked synchronized theta activity (Aftanas et al. 2001a), whereas a larger synchronization in the left anterior and bilaterally over the posterior cortical leads to arousal discrimination (Aftanas et al. 2002). Research conducted by Mai et al. (2019) has demonstrated that inhibition of interoceptive network structures (specifically, the frontotemporal anterior insula network and somatosensory cortices) through repetitive TMS, employing continuous theta burst stimulation, disrupts both the arousal and valence components of emotional processing. Particularly, theta seems to be related to the emotion recognition (ER) process, which corresponds to a person’s ability to effectively manage and respond to an emotional experience. Studies targeting internalizing psychopathologies characterized by disturbances in ER have shown that electrical neurostimulation has a beneficial effect on reported internalizing symptoms (Peña-Gómez et al. 2011, Berlim et al. 2013, Feeser et al. 2014, Conson et al. 2015, McAleer et al. 2023). Moreover, McAleer et al. (2023), using offset theta-tACS, placing electrodes in the two positions between which the highest theta synchrony was recorded at baseline, showed that participants displayed changes in behavioural response with lower reappraise of valence and arousal score, as well as improvements in their clinical score for depression and anxiety (McAleer et al. 2023).

Otherwise, beta frequency has been predominantly observed in sensorimotor cortices and basal ganglia structures, linking its oscillations with changes in the somatosensory processing and motor control (Haegens et al. 2011, Baumgarten et al. 2015, Barone and Rossiter 2021). Recently, it has been shown that beta is also involved in emotion processing related to the Affective Valence dimension of emotions. Studies have observed a potentiation and extension of distributed activation of beta oscillatory responses during the presentation of affective stimuli. Specifically, beta oscillatory responses were higher for unpleasant emotional stimuli in the parietal and occipital areas, and in the occipital area for pleasant emotional stimuli (Güntekin and Basar 2007, Güntekin and Başar 2010).

Our results are in line with previous reports in the literature, showing an involvement of theta oscillatory activity in relation to both the Affective Valence and Arousal dimensions of emotions and, instead, a response of beta oscillatory activity only for the Affective Valence dimension of emotions. Overall, this evidence points to both theta and beta frequencies being engaged during emotional processes, highlighting different aspects of the same phenomenon. In line with previous literature, our data demonstrated the different contributions of these frequencies in the perception of emotions, adding new perspectives on the involvement of the somatosensory system in affective processes. The somatosensory system seems to have a causal role in the perception of cognitive and affective dimensions associated with emotions: neurostimulation of this area has shown effects on the perceptual judgement of emotional contexts in which theta seems to contribute more to cognitive and semantic aspects of the emotion (i.e. deciding whether it is positive or negative and thus lowering arousal) and beta controls the intensity associated to the emotion (i.e. perceiving images as more pleasant). Our study marks an initial endeavour to explore the role of S1 in emotional processes employing tACS, providing preliminary insights into this emerging research perspective and indicating the need for subsequent investigations to further elucidate the implications of S1 involvement in emotional processing. Indeed, the neuromodulatory mechanisms and effects of tACS on emotion processing are still to be unravelled.

Unlike the behavioural data, we found no effect of tACS on physiological responses (i.e. SCR data), which we used more as a control measure of the experimental task. Indeed, the main effect of pleasantness (pleasant versus unpleasant) observed allowed us to find what we expected: a difference between the images used in the experiment. Indeed, the physiological response to positive versus negative images differs, giving credibility to the IAPS’s categorical division of emotional stimuli selected for this study. The absence of evident impact of tACS on autonomic response (e.g. SCR) may be attributed to various factors. First, our electrode montages might have elicited some marginal stimulation of brain regions located near the intended target (e.g. related associative area and motor area), thereby introducing potential confounding variables. Although we cannot be sure that we did not also stimulate areas adjacent to S1, the position of the electrodes was chosen on the basis of precise coordinates taken from previous studies (Holmes and Tamè 2019, Holmes et al. 2019) and customized to each participant’s scalp. Furthermore, the areas adjacent to S1 that may have been stimulated with tACS are nevertheless part of a brain circuit, namely the sensorimotor circuit, which is considered a larger network of which S1 is also a part and involved in various emotional processes (Carr et al. 2003, Leslie et al. 2004, Davis et al. 2017, Quadrelli et al. 2021, Botta et al. 2022). Moreover, such residual stimulation, if present, would have been very low and not focal as for the one that reached the target site. Second, the *a priori* selection of stimulation intensity is a delicate aspect to consider, as it may influence the balance between the magnitude of the potential effect and the successful blinding of participants in experimental procedures; however, previous research reported significant effects using a stimulation intensity of 1 mA peak-to-peak, which is lower than the one we used (i.e. 1.5 mA) (Feurra et al. 2011a, Gundlach et al. 2016a, Antal et al. 2017). Lastly, ongoing debates persist regarding the adequacy of currents applied in low intensity tACS studies in humans to penetrate the skull and modulate brain activity (Liu et al. 2018, Vöröslakos et al. 2018). Nevertheless, the body of behavioural and neural evidence supporting the efficacy of tACS continues to expand (Bikson et al. 2018, Vosskuhl et al. 2018).

Previous studies have found contradictory results on the effect of tACS on physiological responses. For example, a study on emotional regulation by McAleer et al. (2023) observed that offset theta-tACS stimulation reflected an increase in heart rate variability, whereas, in contrast, a study on pain by May et al. (2021) found no effect on autonomic responses (i.e. heart rate and skin conductance fluctuations) using tACS stimulation in the alpha and gamma bands on the PFC and primary somatosensory cortex.

It is conceivable that the correlation between peripheral physiological responses and behavioural reports of valence and arousal may fluctuate with alterations in levels of attention and cognitive processing during the perception of the visual stimuli. Furthermore, it appears that self-reports of arousal do not confer a predictive advantage for electrodermal activity. These self-reports also fail to make a distinctive contribution when valence is included in the model, revealing a dissociative relationship between arousal and valence in relation to physiological arousal (Lang et al. 1993, Kron et al. 2015).

Future research should also consider possible individual differences that can affect the perception of emotional stimuli and then, subsequently, the subjective decision upon them. Indeed, for this study, we only investigated possible individual differences in interoceptive sensitivity using MAIA-2, not including other possible dimensions of interoception (e.g. interoceptive accuracy). Previous studies have demonstrated a relationship between interoceptive accuracy and emotion-related brain activity—especially within the Arousal dimension of emotions and when emotions were experimentally induced using emotional images of facial expressions (Pollatos et al. 2005, Herbert et al. 2007; for a meta-analysis, see Parrinello et al. 2022). In particular, a study by Pollatos et al. (2005) showed that participants with more interoceptive awareness (i.e. better heartbeat perceivers) scored the emotional stimuli significantly more arousing than participants with less interoceptive awareness.

In general, our behavioural findings suggest that the somatosensory system might contribute to the subjective experience of emotion in a more cognitive manner (e.g. by influencing the perception of an emotional stimulus and then expressing a subjective decision) than a purely physiological aspect of feeling. The emergence of novel electrical brain stimulation techniques (e.g. tACS) paves the way to further research scenarios in the relationship between body and emotion, offering new possibilities for investigating, in a non-invasive manner and safely, how electrical stimulation can modulate brain activity related to sensorimotor processing and potentially influence emotional states generation and perception. Additionally, they open up different avenues for exploring the potential therapeutic application of electrical stimulation of the brain in treating emotional disorders through also stimulation of sensorimotor areas, further highlighting the strong link between emotions and the body. Not only we observe an emotion’s motor nature, in which motor aspects can modulate and modify emotional processing (Hajcak et al. 2007, Borgomaneri et al. 2014, 2021, Botta et al. 2022), but also an emotion’s somatotopic nature (Damasio et al. 2000, Craig 2002, Rudrauf et al. 2009, Nummenmaa et al. 2014, 2018, Sel et al. 2014). Indeed, the somatosensory system, stimulated at both mostly sensory-related frequency (i.e. beta) and frequency related to higher emotional and cognitive processes (i.e. theta), seems to show a strong causal relationship with emotion processing, influencing what is seen, heard, and perceived via emotional stimuli. This allows for a shift in focus from a unidirectional conception of the emotion–body relationship to a novel conception of a bidirectional relationship, in which the somatosensory system could be considered a mediator/gatekeeper through which emotions pass.

## Supplementary Material

nsae062_Supp

## Data Availability

Data and all the Supplementary Materials associated with this study have been deposited at https://osf.io/a5c93/.
