# Supplementary material for: The role of the somatosensory system in the feeling of emotions: a neurostimulation study
Source: Soc Cogn Affect Neurosci. 2024 Sep 13;19(1):nsae062. doi: 10.1093/scan/nsae062 (PMC11488518; doi:10.1093/scan/nsae062)
Supplement: nsae062_Supp [file nsae062_supp.zip › scan-24-016-File002.docx]

**SUPPLEMENTARY MATERIALS: The role of the Somatosensory system in the feeling of emotions: a neurostimulation study**

Michelle Giraud^1,2*^, Amir-Homayoun Javadi^2^, Carmen Lenatti^2^, John Allen^2^, Luigi Tamè^2*†^ & Elena Nava^1†^

^1^Department of Psychology, University of Milano-Bicocca, Piazza dell’Ateneo Nuovo 1, 20126, Milano, Italy

^2^School of Psychology, University of Kent, Canterbury, UK

^†^Share senior authorship

***Corresponding author:**

Luigi Tamè

email: L.Tame@kent.ac.uk

Michelle Giraud

email: [m.giraud@campus.unimib.it](mailto:m.giraud@campus.unimib.it)

**Table 1*.*** *IAPS pictures, listed by catalogue number, used in Before and During tACS stimulation with the means and standard deviations arousal of the groups of IAPS items used in the study. A) IAPS pictures selected for female participants. B) IAPS pictures selected for male participants*

| A) CATEGORIES | IAPS images numbers | |
| --- | --- | --- |
| For Female participants | Before-tACS | During-tACS |
| *UHA* (unpleasant highly arousing images) | 3030; 6540; 6563; 3120; 9400; 3195; 9322; 2981; 9183; 3019; 3301; 9571; 9163; 9413; 6350.  **Arousal Mean 6.75 (SD 0.59)** | 2703; 3071; 6510; 3230; 9412; 9184; 9420; 3101; 9301; 9040; 9326; 9570; 9185; 9940; 9635.1.  **Arousal Mean 6.41 (SD 0.55)** |
| *ULA* (unpleasant low arousing images) | 9220; 2900.1; 2695; 9830; 2750; 2455; 9041; 9331; 9832; 9291; 9330, 9001; 9831; 9471; 6800.  **Arousal Mean 4.61 (SD 0.38)** | 2205; 9000; 3300; 9342; 2753; 2301; 9280; 2490; 9290; 9404; 6241; 2722; 9101; 2590; 7078.  **Arousal Mean 4.34 (SD 0.42)** |
| *PHA* (pleasant highly arousing images) | 5621; 1440; 2075; 2216; 8200; 8380; 8496; 1811; 2209; 7330; 5825; 2550; 2045; 2058; 2165.  **Arousal Mean 5.72 (SD 0.64)** | 8370; 8420; 8470; 8190; 7502; 2160; 5910; 2071; 5833; 2150; 2347; 2080; 1710; 2050; 2040.  **Arousal Mean 5.57 (SD 0.57)** |
| *PLA* (pleasant low arousing images) | 5010; 5471; 2381; 5611; 7185; 7006; 7004; 7950; 7100; 5740; 5390; 2037; 7900; 5720; 1450.  **Arousal Mean 2.71 (SD 0.38)** | 5000; 7080; 2038; 5551; 7052; 7187; 7026; 7490; 2620; 5726; 5800; 5020; 1910; 5870; 5711.  **Arousal Mean 2.64 (SD 0.24)** |

| B) CATEGORIES | IAPS images numbers | |
| --- | --- | --- |
| For Male participants | Before-tACS | During-tACS |
| *UHA* (unpleasant highly arousing images) | 3030; 9570; 6540; 3101; 8485; 9040; 2683; 2703; 3500; 9940; 9187; 9300; 9322; 9412; 3019.  **Arousal Mean 6.21 (SD 0.59)** | 3071; 9185; 6550; 3120; 9902; 9635.1; 3195; 9413; 3530; 9910; 9183; 9184; 9325; 2981; 3250.  **Arousal Mean 6.05 (SD 0.53)** |
| *ULA* (unpleasant low arousing images) | 2520; 9830; 9390; 9472; 2301; 2312; 9330; 9031; 9000; 9295; 9090; 2141; 2750; 2722; 2799.  **Arousal Mean 4.65 (SD 0.30)** | 2590; 9280; 9110; 9001; 2900; 2490; 2205; 7380; 9220; 9340; 2206; 9421; 2375.1; 6010; 9007.  **Arousal Mean 4.58 (SD 0.38)** |
| *PHA* (pleasant highly arousing images) | 8185; 8492; 4000.1;4652; 4225; 4085; 4660; 4255; 8179; 8180; 5950; 8080; 8260; 5629; 8163.  **Arousal Mean 7.12 (SD 0.40)** | 5621; 8499; 4659; 4608; 4668; 4180; 4220; 4250; 8178; 8186; 8501; 8170; 8400; 8030; 4007.  **Arousal Mean 7.11 (SD 0.36)** |
| *PLA* (pleasant low arousing images) | 1603; 7080; 5020; 5750; 2370; 2341; 1670; 5202; 5711; 5800; 2235; 2304; 5891; 1605; 1610.  **Arousal Mean 3.26 (SD 0.32)** | 1604; 7900; 5200; 5760; 2501; 2035; 1812; 5779; 5720; 5726; 2314; 2560; 5551; 1900; 1620.  **Arousal Mean 3.11 (SD 0.42)** |

1. *Individual differences: MAIA questionnaire*

The Multidimensional Assessment of Interoceptive Awareness – Version 2 (MAIA-2) is an 8-subscale state-trait self-report questionnaire to measure multiple dimensions of interoception (awareness of bodily sensations) (Mehling *et al.*, 2012; Mehling *et al.*, 2018). The MAIA consists of 8 scales addressing five dimensions of body awareness: Noticing (Awareness of Body Sensations); Not-Distracting and Not-Worrying (Emotional Reaction and Attentional Response to Sensations); Attention Regulation (Capacity to Regulate Attention); Emotional Awareness, Self-Regulation and Body Listening (Awareness of Mind-Body Integration) and Trust (Trusting Body Sensations).

Simple linear regressions were performed to check whether questionnaire scores across scales were significantly related to the Arousal and Affective Valence ratings performed during the tasks. We did not find any significant prediction of the individual MAIA scores on the participant's performance during the experiment, except for the Noticing scale for which the result of the regression indicated the predictor (i.e., Noticing scale) explained the 7% of the variance in Affective Valence participants ratings (F(1,58)=4.69, p=.035), the Noticing scale significantly predicted Affective Valence participants ratings β =0.27, t(58)= 2.16, SE=0.45, p=.035); the Self-regulation scale for which the result of the regression indicated the predictor (i.e., Self-regulation scale) explained the 9% of the variance in Affective Valence participants ratings (F(1,58)=5.99, p=.017), the Self-regulation scale significantly predicted Affective Valence participants ratings β =0.31, t(58)= 2.45, SE=0.65, p=.017); and the Non-distracting scale for which the result of the regression indicated the predictor (i.e., Non-distracting scale) explained the 6% of the variance in Arousal participant’s ratings (F(1,58)=4.15, p=.046), ), the Non-distracting scale significantly predicted Arousal participants ratings β =- 0.26, t(58)= -2.05, SE=0.2, p=.046).
